# Photoperiod-sensitivity genes shape floret development in wheat

**DOI:** 10.1093/jxb/ery449

**Published:** 2018-12-21

**Authors:** Thomas I Pérez-Gianmarco, Gustavo A Slafer, Fernanda G González

**Affiliations:** 1 Department of Crop and Forest Sciences, and AGROTECNIO (Center for Research in Agrotechnology), University of Lleida, Lleida, Spain; 2 CITNOBA, CONICET-UNNOBA. Pergamino, Buenos Aires, Argentina; 3 ICREA (Catalonian Institution for Research and Advanced Studies), Spain; 4 EEA Pergamino INTA. Pergamino, Buenos Aires, Argentina

**Keywords:** Floret development, floret initiation, floret survival, photoperiod sensitivity, *Ppd-1* genes, wheat

## Abstract

Lengthening the pre-anthesis period of stem elongation (or late-reproductive phase, LRP) through altering photoperiod sensitivity has been suggested as a potential means to increase the number of fertile florets at anthesis (NFF) in wheat. However, little is known about the effects that the *Ppd-1* genes modulating plant response to photoperiod may have on reproductive development. Here, five genotypes with either sensitive (*b*) or insensitive (*a*) alleles were grown in chambers under contrasting photoperiods (12 h or 16 h) to assess their effects. The genotypes consisted of the control cultivar Paragon (three *Ppd-1b*) and four near-isogenic lines of Paragon with *Ppd-1a* alleles introgressed from: Chinese Spring (*Ppd-B1a*), GS-100 (*Ppd-A1a*), Sonora 64 (*Ppd-D1a*), and Triple Insensitive (three *Ppd-1a*). Under a 12-h photoperiod, NFF in the genotypes followed the order three *Ppd-1b* > *Ppd-B1a* > *Ppd-A1a* > *Ppd-D1a* > *three Ppd-1a*. Under a 16-h photoperiod the differences were milder, but three *Ppd-1b* still had a greater NFF than the rest. As *Ppd-1a* alleles shortened the LRP, spikes were lighter and the NFF decreased. The results demonstrated for the first time that *Ppd-1a* decreases the maximum number of florets initiated through shortening the floret initiation phase, and this partially explained the variations in NFF. The most important impact of *Ppd-1a* alleles, however, was related to a reduction in survival of floret primordia, which resulted in the lower NFF. These findings reinforce the idea that an increased duration of the LRP, achieved through photoperiod sensitivity, would be useful for increasing wheat yield potential.

## Teaser

Higher photoperiod sensitivity in wheat is associated with more fertile florets per spike at anthesis due to a longer floret initiation phase, which results in higher numbers of initiated florets, and to increased floret survival.

## Introduction

The yield potential of wheat (*Triticum aestivum*) is closely related to the number of fertile florets at anthesis (NFF), given that most fertile florets set grains (Fischer, 1983; Siddique *et al.*, 1989; González *et al.*, 2003*b*) and that yield is mostly sink-limited during grain filling (Fischer, 2007; Serrago *et al.*, 2008; González *et al.*, 2014). In contrast to the fate of primordia of leaves and spikelets, not all the initiated floret primordia continue their development to become fertile florets at anthesis (Kirby, 1988; Siddique *et al.*, 1989; Ferrante *et al.*, 2010). Depending on the position of the spikelet within the spike, between 6 to 12 floret primordia per spikelet are initiated (Langer and Hanif, 1973; Sibony and Pinthus, 1988) but usually only 30–45% reach the fertile floret stage (Langer and Hanif, 1973; Kirby, 1988; González *et al.*, 2011). The availability of assimilates for spike growth during the pre-anthesis period of stem elongation, when florets develop, determines floret survival (Fischer and Stockman, 1980; Kirby, 1988; Ghiglione *et al.*, 2008; González *et al.*, 2011; Ferrante *et al.*, 2013).

Increasing the duration of the pre-anthesis period of stem elongation, also known as the late-reproductive phase (LRP, from terminal spikelet to anthesis), through altering photoperiod sensitivity would increase spike growth and the NFF at anthesis (Slafer *et al.*, 2001; Miralles and Slafer, 2007). Studies performed under controlled (Miralles *et al.*, 2000) and field conditions (González *et al.*, 2003*a*, 2005*a*) in which the photoperiod was changed only during the LRP have concluded that a longer LRP allows for greater assimilate acquisition by the spike, leading to increased spikelet fertility through greater floret survival. However, it has also been suggested that possible direct photoperiod effects might account for the fate of certain floret primordia, i.e. beyond the effect it has on spike dry weight at anthesis through modifying LRP duration (González *et al.*, 2003*a*, 2005*a*). More in-depth research has also shown that photoperiod affects the dynamics of floret development: the shorter the photoperiod, the longer the duration of floret development, which allows some labile florets to complete their development, thus increasing spike fertility (Craufurd and Cartwright, 1989; Miralles *et al.*, 2000; González *et al.*, 2003*a*, 2005*b*).

In contrast, our knowledge of the effects on floret development and the NFF of the genes that modulate plant response to photoperiod (*Ppd-1*) is more limited. These genes consist of a homeoallelic series of loci named *Ppd-D1*, *Ppd-B1*, and *Ppd-A1* (McIntosh *et al.*, 2003) that are located in the short arms of chromosome 2 of the D, B, and A genomes, respectively (Scarth and Law, 1983, 1984). While the wild-type, *Ppd-1b*, is associated with a photoperiod-sensitive phenotype, *Ppd-1a* mutations are associated with a photoperiod-insensitive phenotype, i.e. the mutants have accelerated developmental rates under short, non-inductive photoperiods compared to the wild-type (Shaw *et al.*, 2012; Bentley *et al.*, 2013). To the best of our knowledge, there are only two reports of studies on the impact of *Ppd-1* genes on florets, both conducted under field conditions. The first one (González *et al.*, 2005*b*) showed that the presence of the photoperiod-insensitive allele *Ppd-D1a* reduced the number of fertile florets per spike under short days, while *Ppd-B1a* showed no difference when compared to the photoperiod-sensitive alleles. These results were consistent across two different genetic backgrounds (cultivars Cappelle Desprez and Mercia). As the impact of *Ppd-D1* could be explained by variations in spike dry weight at anthesis (SDWa), which was associated with the duration of the LRP, it was hypothesized it may have altered floret survival. The duration of the LRP was altered by *Ppd-B1*, but no impact on SDWa was observed, resulting in no response in fertile florets per spike when compared to photoperiod-sensitive lines. The second report (Prieto *et al.*, 2018) explored more combinations of *Ppd-1a* alleles, including *Ppd-A1a* for the first time. This study showed that, in general, the presence of *Ppd-1a* alleles reduced floret fertility, mainly associated with floret survival, but the results were not fully consistent between growing seasons.

As far as we are aware, our current study is the first one to be aimed at assessing the effect of *Ppd-1a* alleles on reproductive development, namely the dynamics of floret development and the number of fertile florets at anthesis, under contrasting photoperiods and controlled growth conditions. We aimed to determine whether increasing the insensitivity to photoperiod by introgressing *Ppd-1a* alleles would reduce spike fertility through a reduction in the number of floret primordia that reach the stage of fertile florets at anthesis.

## Materials and methods

### Conditions and treatments

Two independent experiments were carried out in growth chambers under controlled conditions. Treatments consisted of factorial combinations of five genotypes of wheat (*Triticum aestivum* L.) with different sensitivities to photoperiod and two contrasting photoperiod regimes. The five genotypes consisted of the control cultivar Paragon and four near-isogenic lines (NILs) with differing compositions of *Ppd-1* alleles (Table 1; for the procedure to obtain the NILs see Bentley *et al.*, 2013). Either a short (12 h light/12 h dark) or a long (16 h light/8 h dark) photoperiod was applied. Each photoperiod treatment lasted from emergence to anthesis.

**Table 1.** Presence of insensitive alleles (*Ppd-1a*) in the photoperiod-sensitive cultivar Paragon and in near-isogenic lines.

Genotype	Chromosome A	Chromosome B	Chromosome D
Paragon			
P(GS-100-2A)	X		
P(CS-2B)		X	
P(S64-2D)			X
Triple Insensitive	X	X	X

Paragon has *Ppd-1b* alleles in the three genomes. Insensitive alleles were introgressed in the A, B, and D genomes from GS-100, Chinese Spring (CS), and Sonora 64 (S64), respectively.

Pots containing recently sown seeds were placed in a growth chamber that was set with the short photoperiod, and they remained there until the last plant reached anthesis. They were then removed for final sampling, the chamber was reset with the long photoperiod, and a new batch of pots with recently sown seeds was placed in the chamber. To ensure that the same daily total of incident radiation was received for both treatments (~2.36 MJ m^−2^ d^−1^) some of the lights in the chamber were turned off for the long photoperiod treatment. Homogenous light distribution within the growth chamber was assured. The same procedure, i.e. sequential batches of plants grown under short and long photoperiods, was also carried out in a second growth chamber at the same time. Each growth chamber thus represented an independent experiment.

Plants were grown at 16 °C under both photoperiods, in pots (235 ml) irrigated and fertilized with macro- and micro-nutrients. The number of pots per genotype ranged from 38–54 depending on the *Ppd-1* genetic composition and photoperiod treatment: the short photoperiod and the genotypes expected to be more sensitive had more pots than the others, to allow for more sampling. Insects and diseases were prevented by spraying with insecticides and fungicides. Each experiment was arranged as a completely randomized design: all genotypes were equally distributed between chambers and randomly set within them. The pot was considered as the experimental unit. Further details about plant growth conditions can be found in Pérez-Gianmarco *et al.* (2018).

### Measurements, response variables, and analyses

In each experiment, seedling emergence and anthesis were determined by external observation. To determine the terminal spikelet stage, two plants per genotype × photoperiod treatment within each experiment were randomly sampled and dissected under a binocular microscope following the scale of Kirby and Appleyard (1981). Thermal time was calculated assuming a base temperature (*T*_b_) of 0 °C.

For the short photoperiod, during the LRP (from terminal spikelet to anthesis), two plants per genotype within each experiment were also randomly sampled two or three times a week and examined under a binocular microscope to determine the stage of development of all floret primordia within the central spikelet of the spike (Waddington *et al.*, 1983). Floret primordia were named from F1, closest to the rachis, to F*n*, the farthest from the rachis (González *et al.*, 2003*a*). Following each sampling, the remaining plants were re-arranged to keep a canopy-like structure within each growth chamber.

At anthesis, eight plants per genotype within each photoperiod treatment in each experiment were sampled for dissection to count all fertile florets in the spikelets on one side of the spike. The florets were considered to be fertile either when yellow anthers were visible or when the floret score was >9.5 according to the scale of Waddington *et al.* (1983). To estimate the total number of fertile florets per spike (NFF SPK^−1^) this count was multiplied by two and then the florets in the terminal spikelet were added. The number of fertile spikelets per spike (SPKLT_f_ SPK^−1^) was determined by counting the spikelets that bore at least one fertile floret (again using one side of the spike and scaling up). The number of fertile florets per fertile spikelet (NFF SPKLT_f_^−1^) was then calculated as NFF SPK^−1^/SPKLT_f_ SPK^−1^. After all the counts were completed, spikes were oven-dried at 65–70 °C to determine spike dry weight at anthesis (SDWa).

The rate of floret development was calculated for florets that reached the fertile stage in all treatments (the first and second florets from the rachis in the central spikelet, F1 and F2, respectively) as the inverse of thermal time elapsed from stages W4 to W10 (Waddington *et al.*, 1983). Floret initiation dynamics were assessed using linear regression of the form *y=a+bx* relating the number of living florets at a particular time to the thermal time elapsed from seedling emergence. The rate of floret initiation in the central spikelet (RFIc) was estimated as the slope (*b*) of that relationship. The duration of floret initiation in the central spikelet (DFIc) was estimated as the time elapsed from the appearance of the first floret primordium until the maximum number of initiated florets was achieved (NIFc). The timing of the appearance of the first primordium was calculated using the linear regression equation as the value of *x* when *y*=1. The number of fertile florets in the central spikelet (NFFc) was calculated as the mean number of fertile florets determined at anthesis in the central spikelet of each spike. Floret survival (FSc) was calculated as the percentage of the initiated florets (NIFc) that continued developing normally to reach the fertile floret stage at anthesis (NFFc). The number of fertile florets per spike (NFF SPK^−1^) was related to SDWa using a non-linear regression of the form *y=a+bx*^−0.5^ using the TableCurve software (https://systatsoftware.com/, last accessed 22 December 2018). Correlations between response variables were assessed using Pearson’s coefficient. ANOVAs were performed to assess differences among genotypes, photoperiod treatments, experiments, and their double- and triple-interactions. When significant differences were detected, Tukey’s test (α=0.05) for means comparison was performed using Infostat (http://www.infostat.com.ar, last accessed 22 December 2018).

## Results

The genotypes that we used had been shown previously to have a range of LRP durations and SPKLT_t_ SPK^−1^ values according to their *Ppd-1* compositions and the photoperiod conditions (Pérez-Gianmarco *et al.*, 2018). LRP was shortened by ~660 °C d by the triple-stacking of *Ppd-1a* alleles under a short photoperiod (i.e. comparing the three-*Ppd-1b* Paragon with Triple Insensitive), while under a long photoperiod the magnitude of the effect was reduced to ~150 °C d. Consistent with this, Triple Insensitive produced about five fewer spikelets per spike than Paragon under a short photoperiod, while the difference between the two genotypes was reduced to about one spikelet under a long photoperiod. In general, the stronger the insensitivity and the longer the photoperiod, the shorter the LRP and the lower the number of spikelets (for further details see Pérez-Gianmarco *et al.*, 2018). In this context, the aim of the current study was to test the overarching hypothesis that increasing the insensitivity to photoperiod by introgressing *Ppd-1a* alleles would reduce spike fertility through reducing the number of floret primordia that reach the stage of fertile florets at anthesis.

The two independent experiments that we conducted yielded similar results except for NFF SPK^−1^ (Table 2), where one experiment resulted in spikes with 8.8% more florets than the other. As the interactions between experiments with photoperiod and genotypes were not significant for any of the measured variables, means across experiments are presented to describe genotype performance under the contrasting photoperiod treatments. For the most photoperiod-sensitive genotype, Paragon, only 31% of the plants reached anthesis under short photoperiod and the rest showed stalled development after reaching the terminal spikelet stage (see details in Pérez-Gianmarco *et al.*, 2018). Therefore, the results presented here are only from those plants that did reach anthesis.

**Table 2.** Mean-square values for main effects and interactions for ANOVAs performed on parameters relating to spikes, spikelets, and florets in the genotype × photoperiod experiments.

Effects	NFF SPK^−1^	SPKLT_f_ SPK^−1^	NFF SPKLT_f_^−1^	SDW_a_
Experiment (Exp)	108.20*	3.60	0.26	1034
Genotype (Gen)	452.85***	24.05 ***	0.82 ***	36 810***
Photoperiod (Pho)	44.96	2.58	1.43 **	1155
Gen*Pho	78.62**	4.42 **	0.20	9020***
Gen*Pho*Exp	35.17	1.99	0.10	1544

NFF SPK^−1^, number of fertile florets per spike; SPKLT_f_ SPK^−1^, fertile spikelets per spike; NFF SPKLT_f_^−1^, number of fertile florets per fertile spikelet; SDW_a_, spike dry weight at anthesis. *P<0.05, **P<0.01, ***P<0.001.

### Number of fertile florets

**Fig. 1.**
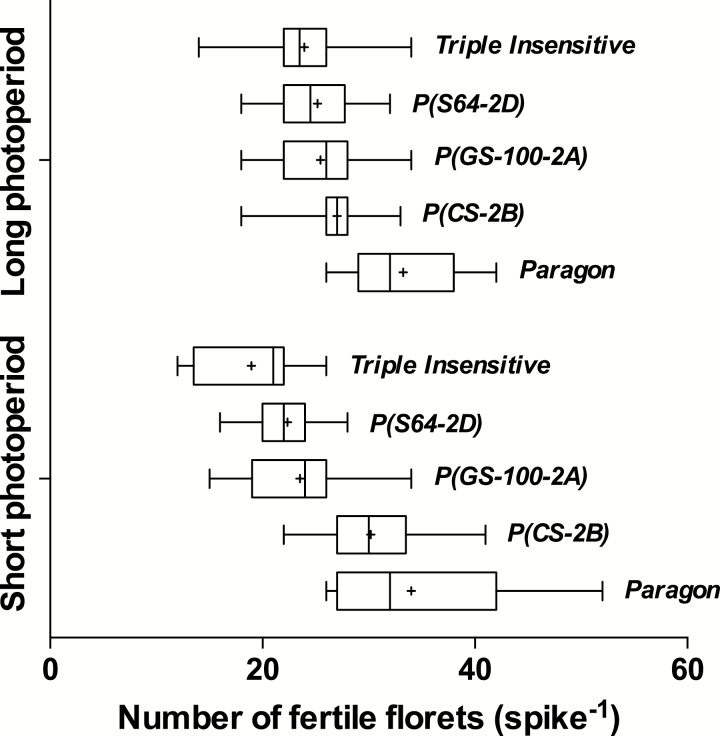
Box-and-whiskers plot for number of fertile florets per spike produced by each genotype under either a long (16-h) or short (12-h) photoperiod. The boxes consist of the 25th to 75th percentiles, with the mean indicated by ‘+’ and the median indicated by the vertical line. The whiskers extend from the minimum to the maximum.

The number of fertile florets per spike (NFF SPK^−1^) depended on the genotype × photoperiod interaction (Table 2). Under the short photoperiod, Paragon reached anthesis with ~35 fertile florets per spike, followed by P(CS-2B), P(GS100-2A), P(S64-2D), and Triple Insensitive, which averaged 30, 24, 22, and 19 fertile florets per spike, respectively (Fig. 1). Under the long photoperiod, genotypes with at least one insensitivity allele carried fewer fertile florets than Paragon, but differences among *Ppd-1a*-bearing lines were not evident. When comparing the most and the least fertile plants for each combination of genotype × photoperiod treatment, the same trends were observed (see the whiskers in Fig. 1).

**Fig. 2.**
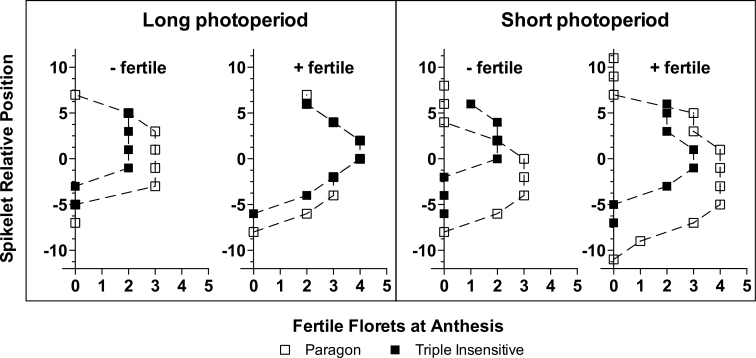
Fertility profiles of most and least fertile spikes (‘+ fertile’ and ‘- fertile’, respectively) for the Paragon and Triple Insensitive genotypes under long (16-h) and short (12-h) photoperiods. The graphs show the number of fertile florets at anthesis for a given spikelet position for one half of the spike, with zero on the *y*-axis representing the central spikelet of the spike.

Variations in NFF SPK^−1^ were highly correlated with SPKLT_f_ SPK^−1^ (*r=*0.96, *P*<0.0001) and with NFF SPKLT_f_^−1^ (*r=*0.88, *P*<0.0001). Under the short photoperiod, Paragon had greater SPKLT_f_ SPK^−1^ and more NFF SPKLT_f_^−1^ (Table 3), achieving a higher NFF SPK^−1^ than the Triple Insensitive line. Most differences in spikelet fertility were observed in the basal and central positions along the spike (Fig. 2). Genotypes with one insensitivity allele had intermediate values for both variables (SPKLT_f_ SPK^−1^, NFF SPKLT_f_^−1^) with P(CS-2B) closer to Paragon, and P(GS-100-2A) and P(S64-2D) closer to Triple Insensitive (Table 3). A similar trend was observed under long photoperiod but with smaller differences among the genotypes. The differences in SPKLT_f_ SPK^−1^ were highly associated with the total number of spikelets per spike (*r=*0.89, *P*<0.0005), as the proportion of total spikelets bearing at least one fertile floret varied between 0.69 and 0.76 without a clear trend among genotypes within the photoperiod treatments.

**Table 3.** Components of the number of fertile florets per spike.

Photoperiod	Genotype	SPKLT_f_ SPK^−1^	NFF SPKLT_f_^−1^
Long	Triple Insensitive	9.4^a^	2.5^bc^
	P(S64-2D)	10.0^a^	2.5^bc^
	P(GS-100-2A)	9.6^a^	2.7^bc^
	P(CS-2B)	10.1^a^	2.7^bc^
	Paragon	11.6^b^	2.7^bc^
Short	Triple Insensitive	9.0^a^	2.1^a^
	P(S64-2D)	9.6^a^	2.3^ab^
	P(GS-100-2A)	9.7^a^	2.4^ab^
	P(CS-2B)	11.4^b^	2.7^bc^
	Paragon	12.3^b^	2.8^c^

SPKLT_f_ SPK^−1^, fertile spikelets per spike; NFF SPKLT_f_^−1^, number of fertile florets per fertile spikelet. Different letters within a column indicate a significant difference between the mean values (Tukey’s test, α*=*0.05).

**Fig. 3.**
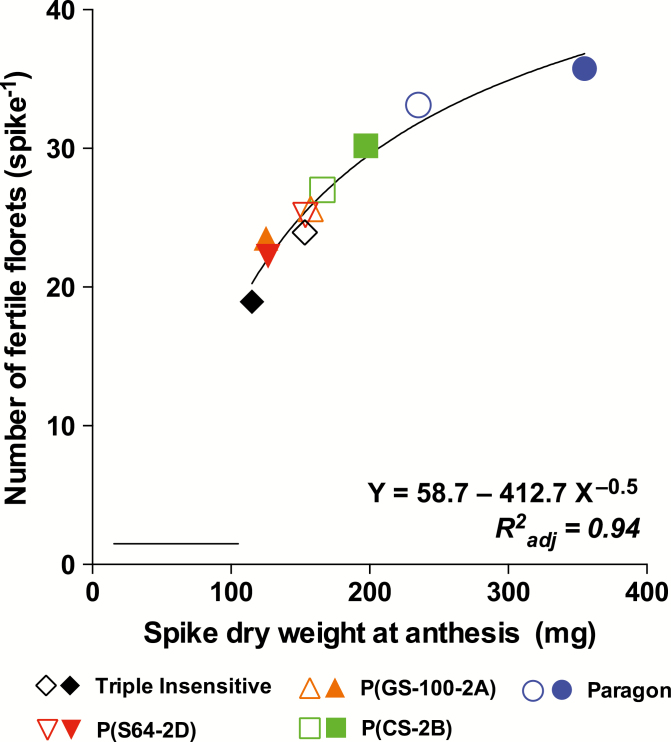
Relationship between the number of fertile florets per spike and the spike dry weight at anthesis for the different genotypes. Open and closed symbols refer to long (16-h) and short (12-h) photoperiods, respectively. The horizontal bar indicates the minimum significant difference for Tukey’s test (α*=*0.05). (This figure is available in colour at *JXB* online.)

Across the entire dataset, 94.3% of the variation in NFF SPK^−1^ was explained by differences in SDWa (Fig. 3). Heavier spikes bore more fertile florets, albeit with NFF SPK^−1^ exhibiting a curvilinear relationship with SDWa. Differences in SDWa were strongly and positively associated with the duration of the LRP (*R*^2^*=*75%, *P*<0.005).

### Floret initiation and survival under a short photoperiod

**Fig. 4.**
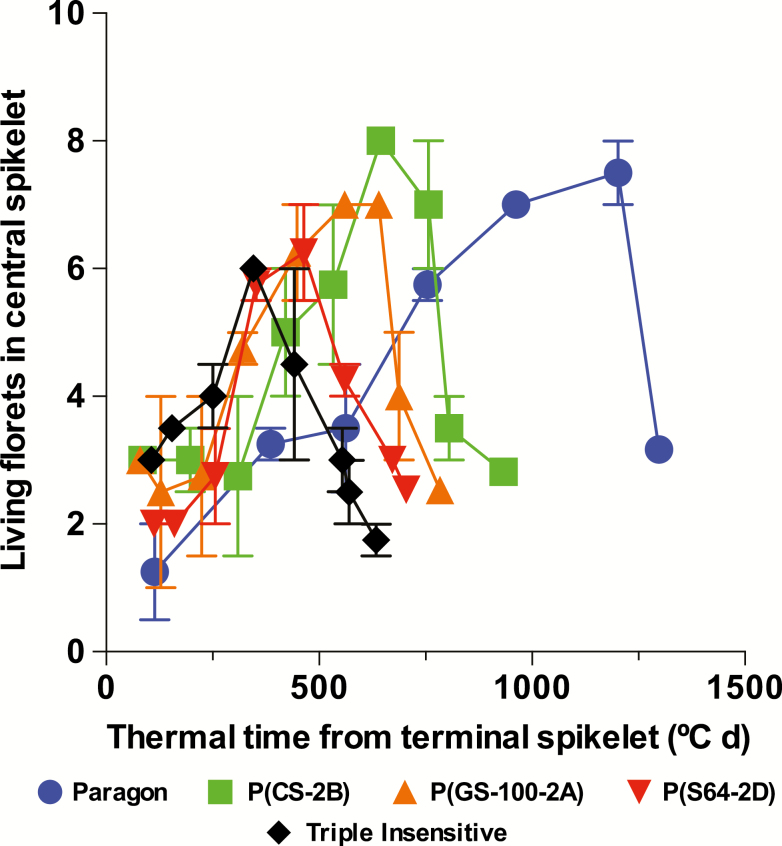
Number of living florets in central spikelets for the different genotypes grown under a short (12-h) photoperiod in relation to thermal time from terminal spikelet. Data are means (±SE) of four replicates. The last data point for each genotype represents the final number of fertile florets in the spikelet. (This figure is available in colour at *JXB* online.)

**Fig. 5.**
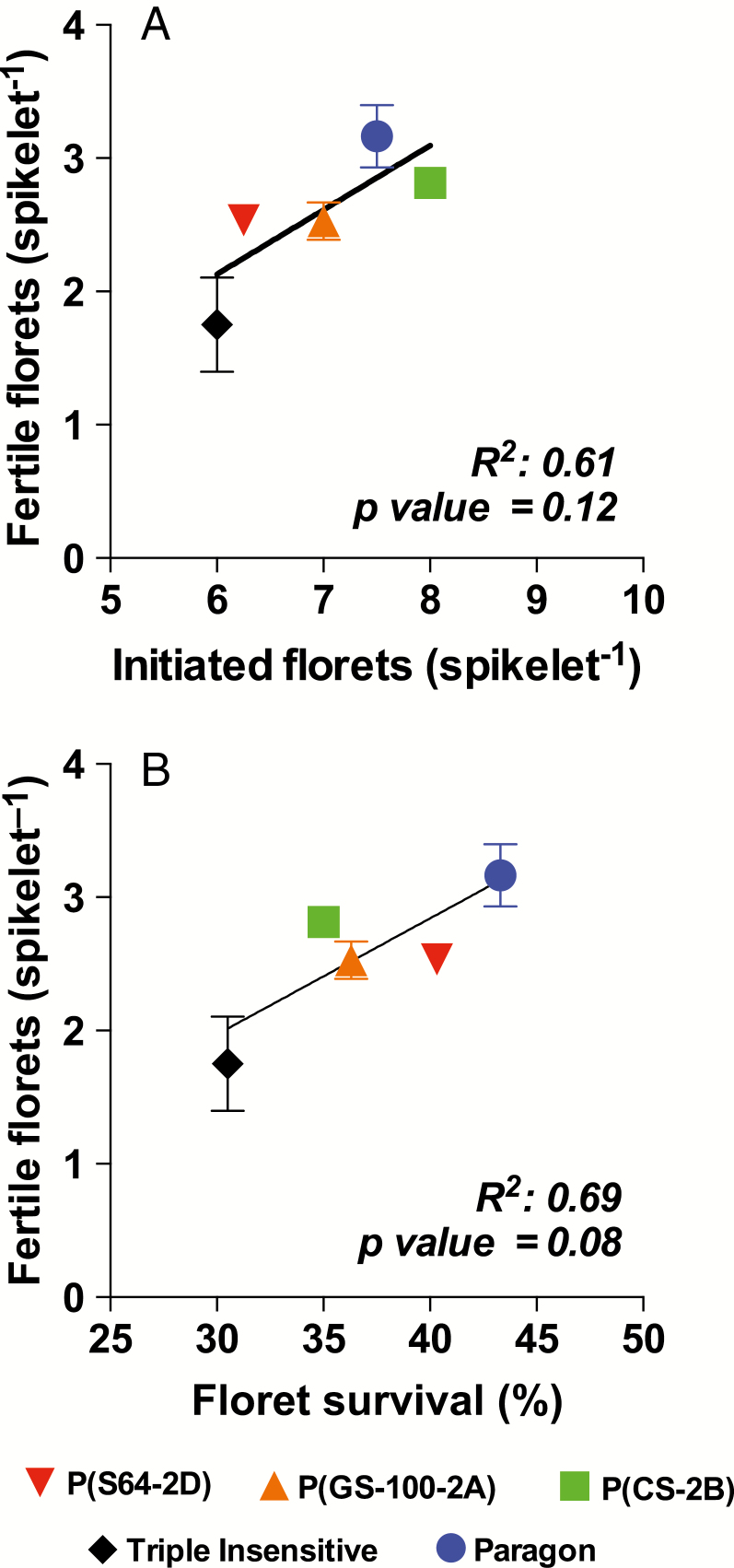
Number of fertile florets in central spikelets for the different genotypes as related to (a) floret initiation, and (b) floret survival. Data are means (±SE) of two replicates. (This figure is available in colour at *JXB* online.)

The number of fertile florets in the central spikelets (NFFc, Table 4) followed the same general pattern that was observed for the average of all fertile spikelets (Table 3). Paragon had a higher NFFc than Triple Insensitive, while the other genotypes showed intermediate values (see last data point for each genotype in Fig. 4). The maximum number of initiated floret primordia in the central spikelets (NIFc) varied from 7.5–8.0 in Paragon and P(CS-2B) to 6.0–6.25 in Triple Insensitive and P(S64-2D), with P(GS-100-2A) having an intermediate value of 7 (Table 4). Floret survival (FSc) was highest in Paragon (43.3%) and lowest in Triple Insensitive (30.0%). P(CS-2B) and P(GS-100-2A) showed intermediate values of ~35%, while for P(S64-2D) it was 40% (Table 4). Thus, as expected, the NFFc was positively associated with floret survival (FSc), although a positive trend was also observed with number of initiated floret primordia (NIFc) (Fig. 5).

**Table 4.** Floret initiation dynamics in the central spikelet under a short photoperiod.

Genotype	NFF_c_	NIF_c_	FS_c_ (%)	RFI_c_ [florets (°Cd) ^−1^]*	DFI_c_ (°Cd)
Triple Insensitive	1.83±0.25	6.00±0.29	30.5	0.0125±0.0021	378
P(S64-2D)	2.52±0.06	6.25±0.41	40.2	0.0149±0.0021	374
P(GS-100-2A)	2.54±0.11	7.00±0.00	36.3	0.0088±0.0014	683
P(CS-2B)	2.80±0.22	8.00±0.48	35.0	0.0094±0.0011	718
Paragon	3.25±0.20	7.50±0.00	43.3	0.0064±0.0005	1101

NFF_c_, number of fertile florets in the central spikelet; NIF_c_, number of initiated florets in the central spikelet; FS_c_, floret survival in the central spikelet; RFI_c_, rate of floret initiation in the central spikelet; DFI_c_, duration of floret initiation in the central spikelet. * For RFI_c_, *R*^2^ of all regressions from which slopes were calculated were greater than or equal to 0.72.

The number of florets initiated can be considered as the result of the duration of the floret initiation phase (DFIc) and the rate at which they are generated (RFIc). Paragon initiated florets over a period of ~1100 °C d while the Triple Insensitive line and P(S64-2D) did so for only about a third of that period (Table 4). Intermediate durations were observed for P(CS-2B) and P(GS-100-2A). RFIc also varied according to the *Ppd-1* composition (Table 4), and was slower for Paragon compared to Triple Insensitive and P(S64-2D), while the other genotypes initiated florets at intermediate rates. Given their strong trade-off, neither DFIc (*r*=0.77, *P*=0.13) nor RFIc (*r*=–0.77, *P*=0.13) by themselves explained the variation in NIFc particularly well. However, while RFIc was almost doubled in Triple Insensitive as compared to Paragon, DFIc was about three times longer in the latter, explaining the difference in NIFc between the two genotypes. In addition, DFIc was highly and positively correlated with LRP across the dataset (*r=*0.97).

### Floret development under a short photoperiod

To more accurately quantify and to further understand the effects of the alleles on the processes of floret initiation and survival, we studied the developmental dynamics of each individual floret primordia within the central spikelets under short days. The performance under long days was not considered further as the differences among the genotypes followed a similar pattern but were of a much smaller magnitude when comparing *Ppd-1a*-bearing genotypes to Paragon, as was expected.

**Fig. 6.**
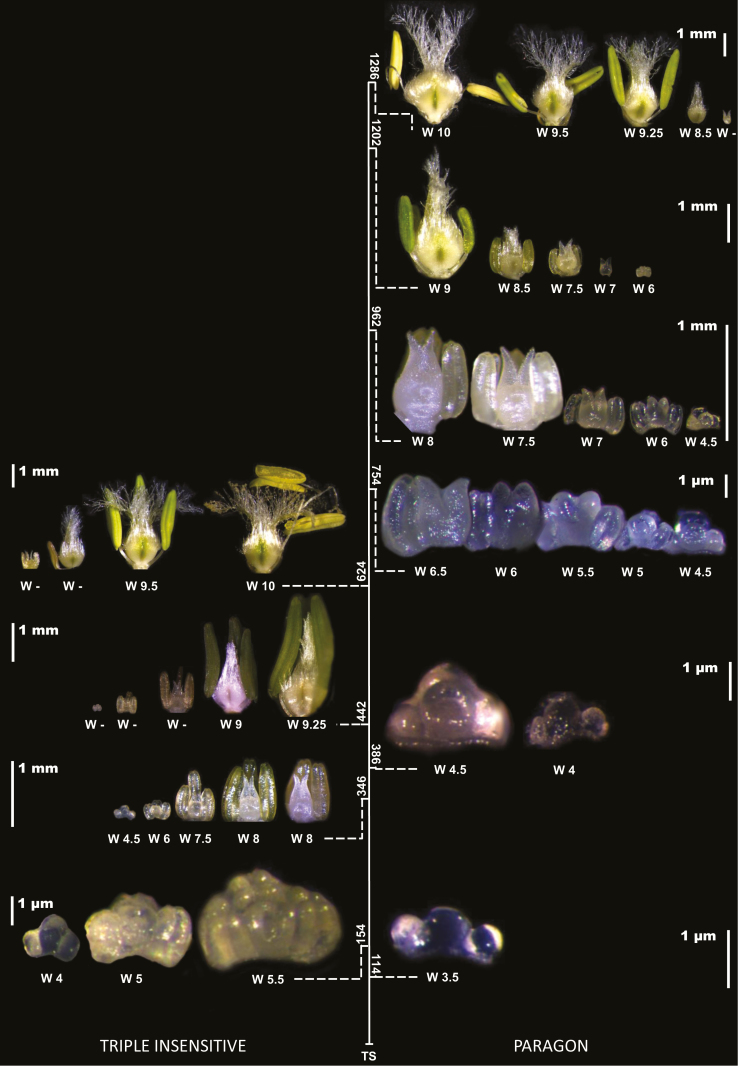
Floret development stages in central spikelets of the Triple Insensitive near-isogenic line (NIL, left) and photoperiod-sensitive Paragon (right) grown under a short (12-h) photoperiod. The vertical scale indicates the thermal time (°Cd) from terminal spikelet to anthesis when each of the images was taken (note the differences in the duration). At each time-point, the florets are arranged in a ‘virtual spikelet’, with the centre of the figure (the thermal-time axis) representing the position of the rachis. The first floret (F1, the most proximal to the rachis) is placed closest to the axis and the fifth floret (F5, when observable) is placed furthest from the axis. Scale bars are shown for each group of florets at a given time-point. (This figure is available in colour at *JXB* online.)

Comparing the two extreme genotypes, Paragon and Triple Insensitive, the impact of the *Ppd-1* genes on the progressive development of floret primordia and the likelihood of some labile florets becoming fertile at anthesis could clearly be seen (Fig. 6). As expected, for each time-point after terminal spikelet (TS) the primordia closest to the rachis were much more developed than those in distal positions within each genotype. For the same floret position at the same time-point, florets were far more developed in the Triple Insensitive than in Paragon. Floret degeneration (when primordia showed signs of incipient dehydration, indicated by the ‘W-’ scale in Fig. 6) occurred more quickly in Triple Insensitive than in Paragon. For example, F3 (the third floret from the rachis) in Triple Insensitive developed normally until stage W7.5 but it then stopped developing and died, whereas in Paragon (with much more time to develop) it continued developing normally to become a fertile floret.

The floret primordia closest to the rachis (F1 and F2) reached anthesis as fertile florets in all genotypes (Fig. 6). Whilst their development did not explain the differences among genotypes in floret fertility, it was still affected by the *Ppd-1a* alleles. The rates of development of both F1 and F2 were faster in the most insensitive genotypes, i.e. the Triple Insensitive line and P(S64-2D), than in Paragon, while the other lines showed intermediate rates (Table 5).

**Table 5.** Rate of development of the first (F1) and second (F2) florets counted from the rachis at the central spikelets of genotypes differing in photoperiod sensitivity grown under a short photoperiod.

Genotype	Rate of floret development [°Cd ×10^2^]^−1^	
	F1	F2
Triple Insensitive	0.179	0.189
P(S64-2D)	0.195	0.223
P(GS-100-2A)	0.152	0.179
P(CS-2B)	0.139	0.132
Paragon	0.101	0.110

Rate of development was calculated as the reciprocal of the thermal-time elapsed from Waddington stages W4 to W10 (Waddington *et al.*, 1983).

**Fig. 7.**
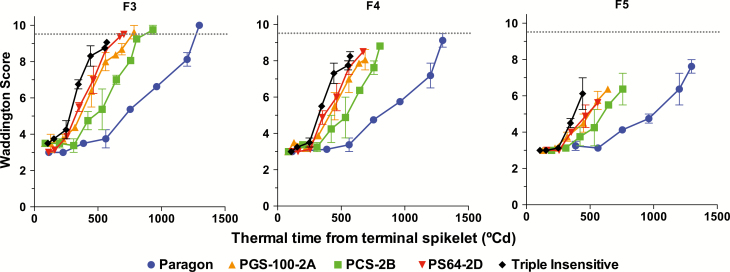
Development of florets (Waddington score) with thermal time for the different genotypes during the late-reproductive phase for the third (F3), fourth (F4), and fifth (F5) florets counted from the rachis of the central spikelets of each genotype grown under a short photoperiod (12 h). Individual florets are plotted from the moment at which they became distinguishable through to anthesis. The dotted line indicates the stage of development at which we considered a floret to be fertile at anthesis. For each curve, the last data point represents the most advanced stage of development that particular floret primordium reached. Data are means (±SE) of four replicates. (This figure is available in colour at *JXB* online.)

Genotypic differences in floret fertility were mainly dependent on the fate of the third and fourth florets, whose rates of development depended on the allelic composition of the genotype (Fig. 7). At anthesis, the third floret (F3) was already fertile in Paragon, and was destined to be fertile in all genotypes except the Triple Insensitive line. The fourth floret (F4) was destined to be fertile only in a few plants of Paragon, whilst carrying at least one *Ppd-1a* allele inhibited the development of this floret and prevented it from proceeding normally and reaching the fertile stage. The effect of *Ppd-1a* alleles on floret development could be also seen in the fifth floret (F5), which did not reach the fertile stage in any of the genotypes, but developed more in Paragon than in the NILs with insensitivity alleles.

**Fig. 8.**
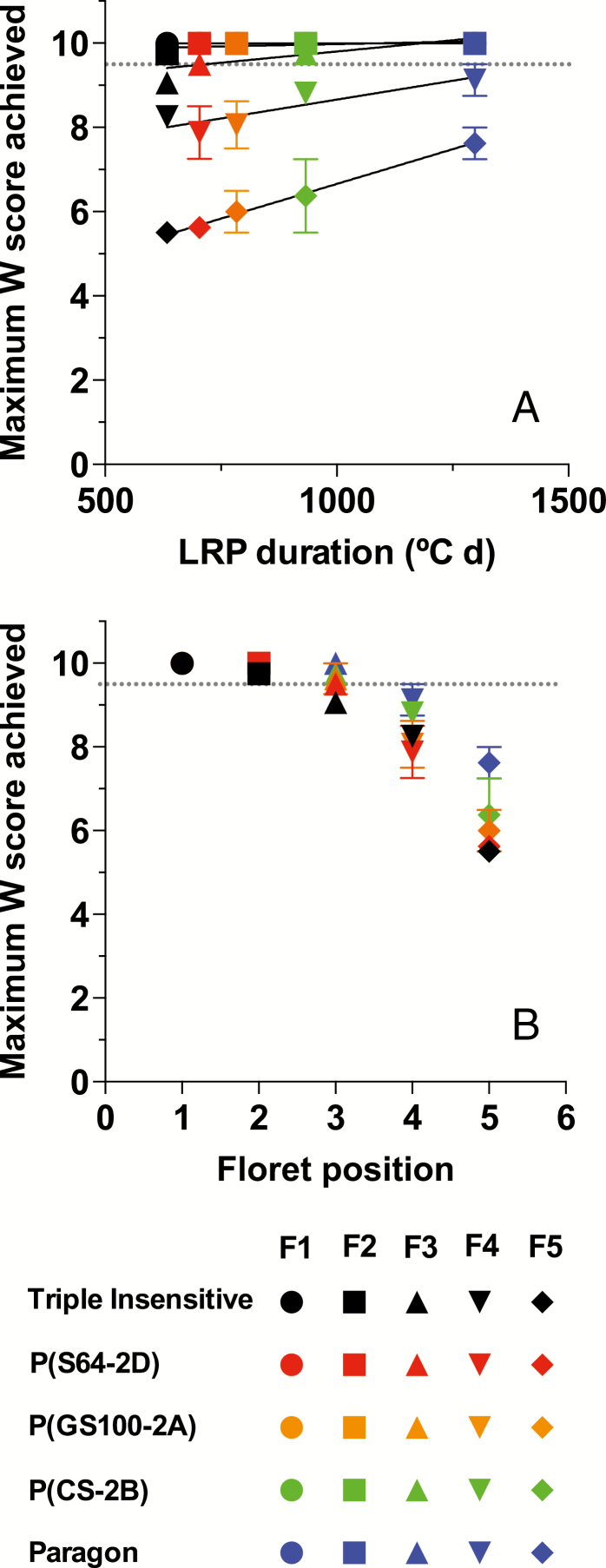
Maximum floret development (Waddington score) achieved by the first (F1) through to the fifth (F5) floret for each genotype grown under a short photoperiod (12 h) as related to (a) LRP duration, and (b) the floret position relative to the rachis. Data are means (±SE) of two replicates.

Therefore, regardless of the ultimate fate of the floret primordia a clear trend was distinguishable for the maximum Waddington score reached by the florets. As the duration of the LRP increased due to the photoperiod sensitivity of the genotypes (from Triple Insensitive to Paragon), the Waddington score for each floret at the end of its development also increased (Fig. 8A). The effect was almost negligible for F1 and F2, beginning to become apparent for F3, and could be clearly seen for F4 and F5. Consequently, the more distal the position from the rachis, the more noticeable was the effect of the *Ppd-1a* alleles on the maximum score achieved by each of the florets (Fig. 8B).

## Discussion

Our results showed that under a short photoperiod, the number of fertile florets per spike at anthesis was associated with the *Ppd-1* composition of the genotype, following the order three *Ppd-1b* (Paragon) > *Ppd-B1a* (Chinese Spring) > *Ppd-A1a* (GS-100) > *Ppd-D1a* (Sonora 64) > three *Ppd-1a* (Triple Insensitive) (Fig. 1). Although the triple-insensitive NIL showed the most extreme insensitivity, the effects of single alleles were never additive: their triple stacking seemed to reach a maximum close to the effect of *Ppd-D1a* alone. It is very likely that, as previously suggested by Shaw *et al.* (2012), there is saturation of the response to insensitive alleles, as seen on phasic development and early ontogeny (Pérez-Gianmarco *et al.*, 2018). The ranking of magnitude of insensitive alleles observed in our study agrees with that reported by Prieto *et al.* (2018) during their first season of experiments using the same genetic material under natural photoperiod (in their second season differences among lines were not clear). Working with isogenic lines on the cultivar Mercia and single-chromosome recombinants lines on Cappelle Desprez, González *et al.* (2005*b*) also observed that the most sensitive genotypes (three *Ppd-1b* alleles) achieved the highest number of fertile florets (NFF), and that NFF decreased when *Ppd-D1a* was introgressed; however, they found no impact of *Ppd-B1a* on NFF. González *et al.* (2005*b*) also reported that differences among lines were partially reduced under a long photoperiod, with no differences when *Ppd-B1a* (also from Chinese Spring) was present. However, in that work, the natural photoperiod was extended only from terminal spikelet onwards, i.e., only during the LRP, which contrasts with our experiments in which the photoperiod was kept constant at 16 h from seedling emergence to anthesis.

Differences in NFF were correlated with the number of fertile spikelets per spike and with the number of fertile florets per fertile spikelet (Fig. 2), similar to the results obtained by Prieto *et al.* (2018). The duration of the late-reproductive period (LRP) was greatest when the three *Ppd-1b* alleles were present (Paragon), as has previously been observed under a short photoperiod (Pérez-Gianmarco *et al.*, 2018) as well as under field conditions with natural photoperiod (Ochagavía *et al.*, 2017). The extension of the LRP brought about increases in the spike dry weight at anthesis (SDWa; Table 2), as has been observed previously (González *et al.*, 2005*b*; Pérez-Gianmarco *et al.*, 2018), which in turn lead to higher values for NFF. As *Ppd-1a* alleles shortened the duration of the LRP, the spikes were lighter and the number of fertile florets was reduced. González *et al.* (2005*b*) also observed a positive relationship between the number of fertile florets and the spike dry weight when different *Ppd-1* alleles were tested, but the relationship was linear whereas the one that we found was curvilinear (Fig. 3). However, our curvilinear relationship reflected the particular response of Paragon to an exceptionally short photoperiod during the LRP (this phase is hardly ever naturally exposed to such short photoperiods for its entire duration under field conditions), in which the architecture of the spikes was altered, with an increase in the length of the rachis between spikelets (see Pérez-Gianmarco *et al.*, 2018). Hence, the heavier spikes in Paragon were not matched by a linear increase in fertile florets because a proportion of the available assimilates were partitioned to rachis instead of florets.

Most previous studies have modified LRP by exposing plants to different photoperiods during that particular phase, and have concluded that the effect on number of fertile florets is largely mediated by the impact on assimilate availability and floret survival associated with spike growth (see González *et al.*, 2011 and references therein). A longer duration of floret initiation is often compensated for by a lower developmental rate, making for small (if any) differences in the maximum number of florets initiated (Craufurd and Cartwright, 1989; Miralles *et al.*, 2000; González *et al.*, 2005*a*). The only previous study examining the impact of different combinations of *Ppd-1* genes on floret development, for NILs sown under field conditions, also found that differences in the number of fertile florets were the result of floret survival, without differences in the number of floret primordia initiated (Prieto *et al.*, 2018). Our results, testing the effects of *Ppd-1* under a constant, short (12-h) photoperiod agreed in general with these findings, as the negative impact of photoperiod insensitivity on the number of fertile florets was related to the effect of *Ppd-1a* alleles on floret survival, which was associated with spike growth (Table 4, Fig. 5B).

However, we also found an impact of *Ppd-1* composition on the maximum number of initiated florets, which was positively associated with the number of fertile florets (Table 4, Fig. 5A). A longer duration of floret initiation, associated with and also taking place during a longer LRP (albeit at a lower rate of floret development), allowed a higher number of florets to be initiated, at least when comparing the extremes of photoperiod-sensitivity alleles (Paragon versus Triple Insensitive). Differences between our results and previous studies may be explained by the fact that in our experiments this phase was subjected to a shorter photoperiod that could possibly be examined in the field given normal sowing dates. Thus, we found almost 3-fold differences among genotypes in duration of floret initiation in the present study, while Prieto *et al.* (2018) previously found only marginal differences. Other studies in which the photoperiod was changed only during the LRP found some direct photoperiod effects on survival of the most distal florets (González *et al.*, 2003*a*, 2005*a*), which had no influence in the final number of fertile florets. Thus, to the best of our knowledge, our present study is the first to report a direct effect of *Ppd-1* alleles on the maximum number of initiated florets. Nevertheless, there are two other reports in literature that show differences in the maximum number of initiated florets associated with fertile florets at anthesis for genotypes of different origins (Guo *et al.*, 2016) or with different durations of LRP (González-Navarro *et al.*, 2015).

Under long days, smaller differences in the number of fertile florets, floret development duration or rate, as well as floret death were found among the genotypes tested relative to under the short photoperiod (data not shown). This is in line with what Pérez-Gianmarco *et al.* (2018) observed for their phenology, given that sensitive and insensitive genotypes tend to behave similarly under an inductive photoperiod. Further insights into the role of *Ppd-1* in shaping floret development under long days could be gained by assessing not only gain-of-function insensitivity alleles (*Ppd-1a*) but also loss-of-function mutant lines developed in the same background, which have been shown to delay flowering beyond that of the triple-sensitive genotype (Paragon) in different inductive photoperiods (Shaw *et al.*, 2013).

The most accepted conceptual model to date suggests that the number of fertile florets depends on floret survival, which is in turn determined by the allocation of assimilates to the growing spike during pre-anthesis (Fischer and Stockman, 1980; Kirby, 1988; Ghiglione *et al.*, 2008; González *et al.*, 2011). In our study, the different combinations of *Ppd-1* alleles modified developmental processes and hence also growth processes, which are hard to separate (i.e. the duration and rate of floret initiation and the time available for florets to develop and grow). Thus, despite having observed a direct physiological effect of *Ppd-1* on the maximum number of initiated florets (Table 4, Fig. 4), in practical terms, our results indicate that it is the impact of *Ppd-1* on floret survival (associated with spike growth) that may be the useful trait for breeding increased wheat yield potential.

In conclusion, as anticipated, *Ppd-1a* reduced the number of fertile florets at anthesis in wheat by modifying floret survival and spike dry weight. We also found, for the first time, an impact of *Ppd-1a* on the maximum number of initiated florets, which was partially associated with the number of fertile florets. In terms of alleles, the number of fertile florets followed the ranking three *Ppd-1b* (Paragon) > *Ppd-B1a* (Chinese Spring) > *Ppd-A1a* (GS-100) > *Ppd-D1a* (Sonora 64) > three *Ppd-1a* (Triple Insensitive). These findings reinforce the idea that increasing the duration of the late-reproductive phase would result in a higher number of fertile florets.

## Abbreviations

DFIc: duration of floret initiation in the central spikelet
FSc: floret survival in the central spikelet
LRP: late-reproductive phase
NFF: number of fertile florets
NFFc: number of fertile florets in the central spikelet
NFF SPK^−1^: number of fertile florets per spike
NFF SPKLT_f_^−1^: number of fertile florets per fertile spikelet
NIFc: number of initiated florets in the central spikelet
NIL: near-isogenic line
Ppd-1: *Photoperiod-1* genes
RFIc: rate of floret initiation in the central spikelet
SDWa: spike dry weight at anthesis
SPKLTS SPK^−1^: total spikelets per spike
SPKLT_t_ SPK^−1^: fertile spikelets per spike.
